# HIV Transmission Potential and Sex Partner Concurrency: Evidence for Racial Disparities in HIV Risk Among Gay and Bisexual Men (MSM)

**DOI:** 10.1007/s10461-021-03430-6

**Published:** 2021-08-17

**Authors:** Carla Tilchin, Jessica Wagner, Christina M. Schumacher, Khalil G. Ghanem, Matthew M. Hamill, Anne Rompalo, Errol Fields, Carl A. Latkin, Adena Greenbaum, Jacky M. Jennings

**Affiliations:** 1 Department of Pediatrics, Center for Child and Community Health Research (CCHR), Johns Hopkins School of Medicine, 5200 Eastern Avenue, Mason F. Lord Bldg—Center Towers, Suite 4200, Baltimore, MD 21224 USA; 2 Department of Health, Behavior and Society, Johns Hopkins Bloomberg School of Public Health, Baltimore, MD USA; 3 Department of Infectious Disease, Johns Hopkins School of Medicine, Baltimore, MD USA; 4 Baltimore City Health Department, Baltimore, MD USA

**Keywords:** HIV, MSM, Racial disparities, Sexual networks, Sex partner concurrency

## Abstract

We determined whether racial disparities in HIV infection among gay and bisexual men (MSM) may be partially explained by racial differences in the HIV transmission potential (i.e. mixing of people living with HIV and people not living with HIV or of unknown HIV serostatus) and density (i.e. sex partner concurrency) of sexual networks. Data included a behavioral survey, testing for HIV, and an egocentric sexual network survey. Mixed effects logistic regressions were used for hypothesis testing. Black (vs. non-Black) MSM were more likely to not know their partner’s HIV serostatus (21.8% vs. 9.6%). Similar proportions reported sex partner concurrency (67.1% vs. 68.0%). In adjusted analyses, among Black MSM, sex partner concurrency significantly increased the odds of an HIV transmission potential partnership (TPP), and this association was not significant among non-Black indexes. The association between an HIV TPP and sex partner concurrency may help explain persistent racial disparities in HIV prevalence.

## Resumen

Determinamos si las disparidades raciales en infecciones del VIH entre hombres homosexuales y bisexuales (hombres que tienen sexo con hombres) puede ser parcialmente explicado por diferencias raciales en el potencial de transmisión del VIH (es decir, mezcla de personas viviendo con VIH y personas que no viven con VIH o cuyo estado serológico del VIH es desconocido) y densidad (es decir, concurrencia de pareja sexual) de redes sexuales. Los datos incluyeron una encuesta de comportamiento, pruebas para el VIH y una encuesta de redes sexuales egocéntrica. Regresiones logísticas de efectos mixtos fueron usados para la prueba de hipótesis. HSH negros (vs. HSH no-negros) eran más propensos a no saber el estado serológico del VIH de su pareja (21.8% vs. 9.6%). Proporciones similares reportaron concurrencia de pareja sexual (67.1% vs. 68.0%). En análisis ajustados, entre HSH negros, la concurrencia de pareja sexual aumentó significativamente las probabilidades de una asociación potencial de transmisión del VIH (TPP por sus siglas en inglés), y esta asociación no fue significativa entre índices de no-negros. La asociación entre una TPP VIH y concurrencia de pareja sexual puede ayudar a explicar disparidades raciales persistentes en la prevalencia del VIH.

## Introduction

Gay and bisexual men (MSM) continue to bear a disproportionate burden of HIV. In 2018, MSM accounted for 66% of new HIV infections in the United States [1]. Among all MSM, Black MSM (BMSM) are at greatest risk of HIV; at current incidence rates, the lifetime risk for HIV is one in two among BMSM, one in five among Latinx MSM, and one in eleven among white MSM [2].

Attempts to understand racial disparities in HIV incidence have largely focused on individual risk behaviors, but a meta-analysis showed that BMSM (vs. non-BMSM) reported fewer sexual risk behaviors, including fewer male sex partners and less condomless anal intercourse [3]. Contextualizing individual-level behaviors within sexual networks by race may highlight key differences in network risk environments and race-specific patterns of sexual networks that are contributing to racial disparities in HIV incidence [4–6].

HIV transmission requires transmission potential in sexual networks. Transmission potential occurs in sexual partnerships or mixing between people living with HIV with viremia and people at risk of acquiring HIV infection (i.e. HIV negative) [7]. Prior work has suggested that there may be higher transmission potential in BMSM networks (vs. non-BMSM networks) due to increased prevalence of HIV infection among BMSM [1], decreased knowledge of positive HIV serostatus among BMSM [8], decreased communication of HIV serostatus among BMSM [9], decreased viral suppression among BMSM [10], and decreased HIV pre-exposure prophylaxis (PrEP) use among HIV negative BMSM compared to other MSM [11]. Additionally, BMSM have been shown to have more same-race partnerships (i.e. racial homophily) than white or Latinx MSM [5]. Racial homophily, when combined with these factors of serostatus knowledge, communication, and virologic non-suppression, could lead to increased transmission potential within and among the sexual networks of BMSM.

Although this mixing is necessary for HIV transmission, transmission cannot be sustained without sufficient network density [12]. One proxy for network density in sexual networks is sexual concurrency. Sexual concurrency is defined as overlapping sexual partnerships where sexual intercourse with one partner occurs between two or more acts of intercourse with another partner [13]. Sex partner concurrency is one form of sexual concurrency where the index reports that their sex partner is having concurrent sexual relationships while having sex with them. This form of concurrency indicates that the index is likely to be connected to a larger sexual network than the network suggested by the egocentric report of their sex partners alone and may provide a more comprehensive measure of the index and partner’s HIV risk [14]. While a meta-analysis suggests that there are no racial differences in the prevalence of concurrent partnerships comparing BMSM to non-BMSM [3], evidence is mixed on the association between race and concurrency among MSM [15–18] The current study will add to the literature by assessing the association between these two crucial factors—HIV transmission potential and sex partner concurrency—by race.

Additionally, there is limited research on the relationships between partner or partnership level factors and sex partner concurrency. In one study among newly positive MSM in San Diego, California, the odds of sex partner concurrency were higher among partnerships with an individual more than 10 years younger than the participant. [19]. Among BMSM in Chicago, Illinois, there was no relationship between sex partner concurrency and HIV seroconversion, but the odds of HIV seroconversion decreased with every social and/or sexual network member who used PrEP [20]. On the individual level, research has demonstrated that individuals who inject drugs are more likely to report concurrent sex partners than individuals who use non-injection drugs or no drugs [21]. Injection drug use is a well-established risk factor for HIV transmission [22, 23].

Given the limitations of prior research, the objective of this analysis was to determine whether the association between HIV transmission potential and sex partner concurrency differed by race (i.e. Black versus non-Black) in a cohort of MSM in a mid-Atlantic U.S. city.

## Methods

### Study Population

The Understanding Sexual Health in Networks (USHINE) study is a prospective cohort study conducted by an academic-public health partnership in collaboration with the Centers for Disease Control and Prevention to understand the individual, systems, and network-level factors associated with syphilis transmission among MSM in Baltimore City. Participants were recruited from two health department sexual health clinics, a federally qualified health center that focuses on LGBTQ + health, a community-based LGBTQ + organization, community engagement events, and respondent driven sampling (RDS). RDS is a peer referral method used commonly when recruiting hard to reach populations such as commercial sex workers or MSM [24]. Individuals were eligible to participate if they reported male sex at birth, current male gender, age 18–45, sex with a man in the past six months, residence in Baltimore City, and were willing and able to give informed consent for the study. Data for this analysis included participants enrolled from July 20, 2018 to February 28, 2020.

### Study Procedures

Study visits occurred every three months for up to two years. Each visit included biological testing for syphilis, HIV, and three-site chlamydia and gonorrhea testing (e.g. penile, anal, oropharyngeal). Participants also completed a survey using Audio Computer-Assisted Self-Interview (ACASI) software to collect demographic, sexual behavior, substance use, pre-exposure prophylaxis (PrEP), and antiretroviral (ART) use data. To gather data on the participant’s sex partners in the past three months, study staff conducted a face-to-face egocentric sexual network survey with participants, herein referred to as an index, and entered the network data into a database. The interview began with a free list of the first, last, and nicknames of all sex partners from the past three months and partner demographics. Among the subset of the index’s three most recent sex partners, study staff then asked indexes additional questions, including their partner’s HIV serostatus, PrEP status, ART status, and concurrency status. Only data from the baseline visit and three most recent sex partners was used in this analysis except for index ART use which was accidentally omitted from baseline and asked first at the three month follow-up visit. This study was approved by the Johns Hopkins School of Medicine IRB. All participants provided written informed consent.

### Measures

The primary exposure and outcome were measured on the partnership level between the index and their sex partner. The primary exposure was sex partner concurrency, ascertained from the question, “While you were having sex with [partner], was [partner] having sex with other people?” and categorized as probably or definitely no vs. probably or definitely yes. The primary outcome was an HIV transmission potential partnership (TPP). Although the use of person-first language when discussing HIV status (i.e. person living with HIV) helps reduce stigma [25] and research should continuously work to de-stigmatize HIV, for clarity in defining this complex outcome, we will refer to individuals as HIV positive, HIV negative, or HIV unknown. For the purposes of this analysis, an HIV TPP was defined as a partnership between either an HIV positive index with an HIV negative or HIV unknown partner, or an HIV negative index with an HIV positive or HIV unknown partner. A partnership with an individual of unknown HIV status was conservatively categorized as an HIV TPP because the partner’s true status may be serodiscordant with the index’s. If the index reported daily ART use or daily PrEP use in the past month, the partnership was not coded as an HIV TPP regardless of the index or partner’s HIV serostatus. If the index reported their partner was on PrEP or ART in the past three months, the partnership was also not coded as an HIV TPP, regardless of the index or partner’s HIV serostatus. The inclusion of PrEP use in defining an HIV TPP attempts to account for the 99% efficacy of daily PrEP use in the prevention of HIV acquisition [26], and the inclusion of ART use in defining an HIV TPP attempts to account for viral loads below 400 copies/mL as undetectable and untransmissible [27].

Index-level covariates included race, age, number of sex partners, non-injection drug use, injection drug use (IDU), PrEP use, ART use, and baseline HIV and STI status (i.e. syphilis, any site chlamydia and gonorrhea). Race was categorized as Black or non-Black. Age was categorized as young adults (< 25 years) or adults (≥ 25 years). The number of sex partners in the past three months was measured continuously. Non-injection drug use in the past three months was ascertained by the question, “In the past three months, have you used any non-injection drugs (drugs you did not inject) other than those prescribed for you?” and categorized as yes or no. Injection drug use in the past three months was ascertained by the question, “In the past three months, on average, how often did you inject?” and categorized as never vs. ever. Index PrEP or ART use in the past month was assessed separately and categorized as daily PrEP or ART use in the past one month vs. infrequent (less than daily) or no PrEP/ART use in the past one month. HIV serostatus was determined by an HIV rapid test with ELISA confirmation, medical record documentation, or in a few cases participant self-report. Syphilis positivity was defined as a rapid plasma reagin (RPR) titer greater than 1:8 in combination with a positive treponemal test. Chlamydia and/or gonorrhea positivity was determined by a nucleic acid amplification test (NAAT), and individuals were categorized as positive by a positive NAAT at any anatomical site.

All partnership level variables were ascertained from the index. Partnership level covariates included partner age difference, condomless anal intercourse, partner HIV serostatus, partner PrEP use, and partner ART use. Age difference was dichotomized to an age difference greater than ± five years. Condomless anal intercourse at last anal sex was categorized as yes if the index answered no to condom use at last anal sex. Partner PrEP or ART use were ascertained separately by the questions, “Was [partner] taking PrEP/(or separately) HIV medication in the past three months?” with binary response options of yes or no. All variables included item responses for “Don’t know” and “Refuse to answer” and were coded as missing if selected.

### Statistical Testing

Summary statistics were generated to characterize indexes and their partners overall and by index race and separately, by index race and HIV transmission potential. Chi-squared tests and Mann–Whitney tests were used, as appropriate, to test for significant associations. Although the primary outcome was HIV transmission potential at the partnership level, HIV transmission potential was defined at both the index and partnership level. An individual was categorized as having any HIV transmission potential if the index had any (vs. no) HIV TPPs. Unadjusted mixed effects logistic regressions were used to determine the odds of an HIV TPP associated with sex partner concurrency in strata of index race. Mixed effects logistic regression estimates were also adjusted for IDU as we hypothesized IDU might increase both the prevalence of sex partner concurrency and of HIV TPPs [21, 28], and there were significant differences in HIV TPP by IDU among Black indexes. All regression estimates accounted for the fact that partnerships were nested within indexes. Although significant in chi-squared analysis, index and partner HIV serostatuses were not included in regression analyses because they were used to define the primary outcome of HIV transmission risk, and only three HIV transmission risk partnerships were among HIV negative indexes and HIV positive partners. Syphilis positivity was also not included in regression estimates because we hypothesized it was on the causal pathway between sex partner concurrency and HIV transmission risk potential. Statistical significance in bivariate analyses was defined by a p-value < 0.05 and in regression analyses by a 95% confidence interval that did not cross 1.0. All analyses were conducted using Stata 15.0 (Stat Corp, College Station, TX), and the visualization was created using the igraph package in R (Version 3.6.3).

## Results

### Study Population

**Fig. 1:**
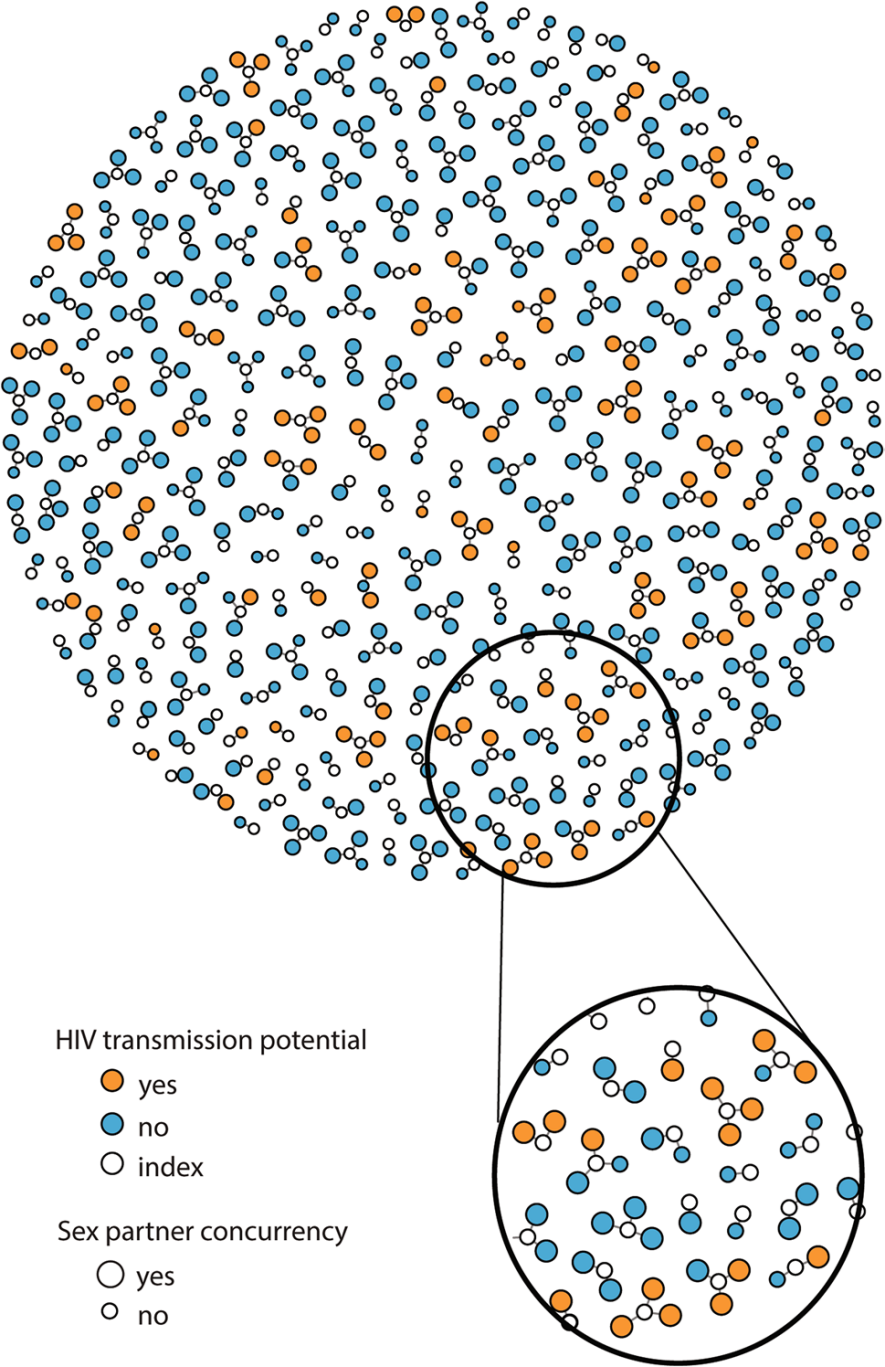
Egocentric network diagram of Black indexes and their most recent (≤ 3) sex partners in Baltimore City, July 2018-March 2020 (N = 586). All indexes are white circles connected to each of their most recent (≤ 3) sex partners. Partners are blue circles if they do not represent an HIV transmission risk partnership and orange circles if they do represent an HIV transmission risk partnership. Partners are small circles if the index did not report that their partner was having concurrent sex while they were having sex with them (sex partner concurrency) and large circles if they did report sex partner concurrency. All indexes are small circles

A total of 567 individuals were screened for the study, of whom 74.4% (n = 422) were eligible. Approximately one percent (n = 5) refused to participate, and 98.8% (n = 417) enrolled and completed a baseline visit. Among these, 96.6% (n = 402) named at least one sexual partner in the past three months, of whom 98.6% (n = 396) had a known HIV serostatus. Among the 396, 93.7% (n = 371) reported sex partner concurrency for at least one partner and were included in this analysis. These 371 indexes reported a cumulative 836 (median: 3, range: 0–3) three most recent sex partnerships. An egocentric network diagram of Black indexes and their three most recent partners demonstrates these partnerships visually (Fig. 1).

On the index level, 72.8% (n = 270) of individuals were Black (Table 1). Overall, 73.3% (n = 272) were ≥ 25 years of age. Indexes overall reported a median of three sex partners in the past three months (IQR = 4.0), 34.8% (n = 129) reported non-injection drug use in the past three months, 8.9% (n = 33) reported IDU in the past three months, and 38.8% (n = 144) were HIV positive. Among HIV positive indexes (n = 144), 33.3% (n = 48) reported daily ART use in the past one month, and among HIV negative indexes (n = 227), 38.8% (n = 88) reported daily PrEP use in the past one month. Overall, 11.3% (n = 42) were syphilis positive, 20.0% (n = 74) were chlamydia and/or gonorrhea positive, and 25.3% (n = 94) were in any HIV TPPs. Black indexes compared to non-Black indexes reported fewer sex partners in the past three months (median = 2, IQR = 3.0, vs. median = 4, IQR = 3.0, p-value = 0.002), were more likely to be living with HIV (45.9% vs. 19.8%, p-value < 0.001), less likely to report daily PrEP use in the past one month (27.4% vs. 59.3%, p-value < 0.001), more likely to be syphilis positive (13.7% vs. 5.0%, p-value = 0.011), and more likely to be in any HIV transmission risk partnerships (28.2% vs. 17.8%, p-value = 0.042). There were no differences in injection or non-injection drug use by index race.

**Table 1:** Characteristics of indexes (participants) (N = 371) and partnerships (N = 836) overall and by index race in the Understanding Sexual Health in Networks Study (USHINE), Baltimore City, July 2018-March 2020

Index Level	Overall N = 371		Black index N = 270		Non-Black index N = 101		p-value
	N	%	N	%	N	%	
Age ≥ 25 years	272	73.3	196	72.6	76	75.3	0.607
> High school education	220	59.3	129	47.8	91	90.1	** < 0.001**
Sex partners, past three months, median (IQR)	3	(4.0)	2	(3.0)	4	(3.0)	**0.002**
Non-injection drug use, past three months	129	34.8	94	34.8	35	34.7	0.940
Injection drug use, past three months	33	8.9	23	8.5	10	9.9	0.692
HIV positive^1^	144	38.8	124	45.9	20	19.8	** < 0.001**
PrEP use, past one month ^2^	88	38.8	40	27.4	48	59.3	** < 0.001**
ART, past one month^2^	48	33.3	42	33.9	6	30.0	0.787
Syphilis positive^3^	42	11.3	37	13.7	5	5.0	**0.011**
Chlamydia and/or gonorrhea positive^4^	74	20.0	53	19.6	21	20.8	0.947
Any HIV transmission potential^5^	94	25.3	76	28.2	18	17.8	**0.042**

Partnership Level	Overall N = 836		Black index partnerships N = 586		Non-Black index partnerships N = 250		p-value
	N	%	N	%	N	%	
Age difference (> ± 5 years)	346	41.4	227	38.7	119	47.6	**0.027**
Condomless anal intercourse, last sex	560	57.0	372	63.5	188	75.2	**0.001**
Partner HIV serotatus^6^							** < 0.001**
HIV negative	562	67.2	358	61.1	204	81.6	
HIV positive	122	14.6	100	17.1	22	8.8	
HIV unknown	152	18.2	128	21.8	24	9.6	
Partner PrEP use, past three months^7^	176	31.3	89	24.9	87	42.7	**0.005**
Partner ART use, past three months^7^	100	82.0	84	84.0	16	72.7	0.584
Sex partner concurrency^8^	563	67.3	393	67.1	170	68.0	0.462
HIV transmission potential (TPP)^5^	160	19.1	132	22.5	28	11.2	** < 0.001**

^1^Determined by an HIV rapid test with ELISA confirmation, medical record documentation, or in a few cases, participant self-report

^2^Defined as daily PrEP/ART use in the past one month vs. infrequent or no PrEP/ART use

^3^Defined as a rapid plasma reagin (RPR) titer greater than 1:8 in combination with a positive treponemal test

^4^Determined by nucleic acid amplification test (NAAT) at any anatomic site

^5^Defined as a partnership between an HIV positive index with an HIV negative or HIV unknown partner or vice versa. If the index reported PrEP or ART use for himself or his partner in the past one month, the partnership was not coded as an HIV transmission potential. At the index level, HIV transmission potential is defined as any vs. none

^6^Determined by index self-report

^7^Determined by index self-report. Partner PrEP status is 32.4% unknown (n = 182) among HIV negative partners, and partner ART status is 16.4% unknown (n = 20) among HIV positive partners

^8^Determined by index self-report of whether their partner was having sex with other people while having sex with them

On the partnership level, 70.1% (n = 586) of partnerships were among Black indexes (Table 1). Overall, 41.4% (n = 346) of partnerships had an age difference greater than ± five years, 57.0% (n = 560) of partnerships reported condomless anal intercourse at last sex, 14.6% (n = 122) of partners were HIV positive, 31.3% (n = 176) of HIV negative partners were on PrEP, 82.0% (n = 100) of HIV positive partners were on ART, and 67.3% (n = 563) of partnerships reported sex partner concurrency. Black indexes compared to non-Black indexes were less likely to be in partnerships with an age difference greater than ± five years (38.7% vs. 47.6%, p-value = 0.027), less likely to report condomless anal intercourse at last sex (63.5% vs. 75.2%, p-value = 0.001), more likely to have an HIV positive partner (17.1% vs. 8.8%, p-value < 0.001), more likely to not know their partner’s HIV serostatus (i.e. HIV unknown) (21.8% vs. 9.6%, p-value < 0.001), less likely to report partner PrEP use (24.9% vs. 42.7%, p-value = 0.005), and more likely to be in an HIV TPP (22.5% vs. 11.2%, p-value < 0.001). Black indexes were no more likely that non-Black indexes to report sex partner concurrency.

### HIV Transmission Potential by Race and Sex Partner Concurrency

Among Black indexes, 28.2% (n = 76) had at least one HIV TPP (i.e. any) and 22.5% (n = 132) of partnerships among Black indexes were HIV TPPs (Table 2). Among non-Black indexes, 17.8% (n = 18) of indexes had at least one HIV TPP and 11.2% (n = 28) of partnerships among non-Black indexes were HIV TPPs (Table 2). Among Black indexes, having at least one HIV TPP (vs. none) was associated with IDU (14.5% vs. 6.2%, p-value = 0.027), positive syphilis status (21.1% vs. 10.8%, p-value = 0.029), and positive HIV serostatus (64.5% vs. 38.7%, p-value < 0.001). Among non-Black indexes, having at least one HIV TPP (vs. none) was also associated with positive HIV serostatus (50.0% vs. 13.3%, p-value < 0.001). Although not statistically significant likely due to small sample sizes, a higher proportion of Black indexes with at least one HIV TPP (vs. none) were IDUs (22.2% vs. 7.2%, p-value = 0.054).

**Table 2:** Characteristics of indexes (participants) (N = 371) and partnerships (N = 836) by index race and HIV transmission potential (TPP) in the Understanding Sexual Health in Networks Study (USHINE), Baltimore City, July 2018-March 2020

	Black index N = 270					Non-Black index N = 101				
	Any HIV TPP N = 76 (28.2%)		No HIV TPP N = 194 (71.9%)			Any HIV TPP N = 18 (17.8%)		No HIV TPP N = 83 (82.2%)		
Index Level	N	%	N	%	p-value	N	%	N	%	p-value
Age ≥ 25 years	59	77.6	137	70.6	0.245	14	77.8	62	74.7	0.784
> High school graduation	23	39.6	106	50.0	0.162	8	80.0	83	91.2	0.260
Sex partners, past three months, median (IQR)	3	(2.5)	2	(3.0)	0.217	3	(3)	3	(4)	0.845
Non-injection drug use, past three months	29	38.2	65	33.5	0.442	7	38.9	28	33.7	0.677
Injection drug use, past three months	11	14.5	12	6.2	**0.027**	4	22.2	6	7.2	0.054
HIV positive^2^	49	64.5	75	38.7	** < 0.001**	9	50.0	11	13.3	** < 0.001**
Syphilis positive^3^	16	21.1	21	10.8	**0.029**	1	5.6	4	4.8	0.837
Chlamydia and/or gonorrhea positive^4^	14	18.4	39	20.1	0.700	2	11.1	19	22.9	0.543

	Black index partnerships N = 586					Non-Black index partnerships N = 250				
	HIV TPP N = 132 (22.5%)		No HIV TPP N = 454 (77.5%)			HIV TPP N = 28 (11.2%)		No HIV TPP N = 222 (88.8%)		
Partnership Level	N	%	N	%	p-value	N	%	N	%	p-value
Age difference (> ± 5 years)	53	40.2	174	38.3	0.259	14	50.0	105	47.30	0.421
Condomless anal intercourse, last sex	78	59.1	294	64.8	0.234	17	60.7	171	77.0	0.060
Partner HIV serostatus^5^					** < 0.001**					** < 0.001**
HIV negative	43	32.6	315	69.4		5	17.9	199	89.6	
HIV positive	1	0.8	99	21.8		2	7.1	20	9.0	
HIV unknown	88	66.7	40	8.8		21	75.0	3	1.4	
Sex partner concurrency^6^	108	81.8	285	62.8	** < 0.001**	21	75.0	149	67.1	0.124

^1^Defined as a partnership between an HIV positive index with an HIV negative or HIV unknown partner or vice versa. If the index reported he or his partner was on PrEP or ARTs, the partnership was not coded as an HIV transmission risk partnership regardless of the partner’s HIV serostatus

^2^Determined by an HIV rapid test with ELISA confirmation, medical record documentation, or in a few cases, participant self-report

^3^Defined as a rapid plasma reagin (RPR) titer greater than 1:8 in combination with a positive treponemal test

^4^Determined by nucleic acid amplification test (NAAT)

^5^Determined by index self-report

^6^Determined by index self-report of whether their partner was having sex with other people while having sex with them

On the partnership level among Black indexes, HIV TPPs (versus non-HIV TPPs) occurred less frequently with an HIV negative partner (32.6% vs. 69.4%) or an HIV positive partner (0.8% vs. 21.8%), p-value < 0.001. Conversely, HIV TPPs (versus non-HIV TPPs) occurred more frequently with a partner of unknown HIV serostatus (66.7% vs. 8.8%, p-value < 0.001). In other words, the majority of HIV transmission potential among Black indexes was driven by partnerships in which a partner’s HIV status was unknown. Importantly, HIV TPPs (verses non-HIV TPPs) occurred more frequently among partnerships with sex partner concurrency (81.8% vs. 62.8%, p-value < 0.001). On the partnership level among non-Black indexes, HIV TPP (versus non-HIV TPP) occurred less frequently with an HIV negative partner (17.9% vs. 89.6%) and occurred more frequently with a partner of unknown HIV serostatus (75.0% vs. 1.4%), p-value < 0.001.

Among Black indexes, there was a 3.24-fold increase in the unadjusted odds of an HIV TPP among partnerships with sex partner concurrency (95% CI: 1.65, 6.37, p-value = 0.001). Among non-Black indexes, there was no difference in the odds of an HIV TPP by sex partner concurrency (OR: 2.58, 95% CI: 0.79, 8.43, p-value = 0.116). Because patterns of IDU differed by HIV TPP in both Black and non-Black participants, regression estimates were adjusted for IDU. In adjusted analyses, among Black indexes there was a 2.97-fold increase in odds of an HIV TPP among partnerships with sex partner concurrency (95% CI: 1.49, 5.89, p-value = 0.002), and among non-Black indexes, there remained no significant difference in the odds of an HIV TPP by sex partner concurrency (aOR: 2.69, 95% CI:0.84, 8.62, p-value = 0.10). Additionally, adjusting regression estimates by age did not change regression estimates meaningfully.

## Discussion

Among Black index partnerships, we found evidence of the necessary and sufficient causes for HIV transmission, including HIV transmission potential (i.e. mixing between individuals living with HIV with viremia and HIV negative or HIV unknown individuals) combined with dense sexual network structures (i.e. sex partner concurrency). We did not find this evidence among non-Black index partnerships. Crucial to these findings is that while there was no difference between Black and non-Black indexes in the proportion of partnerships with sex partner concurrency, there was 2.97-fold increase in odds that a partnership with sex partner concurrency was an HIV TPP among Black indexes. Visually, this association can be observed by the small amount of small orange dots (which represent an HIV TPP without partner concurrency) in Fig. 1. While sex partner concurrency was measured and is described here at the partnership level, sex partner concurrency impacts the risk of transmission across the connected component (or network), including beyond the three most recent partners depicted visually in Fig. 1.

This association between sex partner concurrency and HIV TPP remained largely unchanged when estimates were adjusted for IDU, suggesting that these relationships are independent of injection drug use. The association between sex partner concurrency and HIV TPP was not statistically significant among non-Black indexes. In post-hoc power calculations, there was 74% power to detect the hypothesized relationship among non-Black index participants at a significance level of 0.05. The association between these two crucial factors at the partnership level—HIV transmission potential and sex partner concurrency—may help explain persistent racial disparities in HIV prevalence.

Among MSM in New York City, Tieu et al. found that among HIV positive indexes, individual concurrency was associated with 2.08 (95% CI: 1.02, 4.21) increased odds of serodiscordant/serounknown condomless anal or vaginal intercourse (SDUI), and among HIV negative indexes, sex partner concurrency was associated with 2.43 (95% CI: 1.28, 4.63) increased odds of SDUI [15]. We advance this knowledge by incorporating PrEP and ART status in categorizations of HIV transmission risk, delineate between serodiscordant and serounknown partnerships, and investigate these relationships separately by race to determine racial differences in these relationships.

Similar to prior research, Black indexes compared to non-Black indexes reported fewer sexual risk behaviors, including fewer sex partners in the past three months, fewer partnerships where the partnership age difference was greater than ± five years, and less condomless anal intercourse at last sex [3, 4]. HIV transmission potential was largely comprised of partners of unknown HIV serostatus among both Black and non-Black indexes, but Black indexes (vs. non-Black) were more likely to not know the HIV serostatus of their partner (21.8% vs. 9.6%). This adds to the inconclusive evidence of the impact of unknown HIV serostatus partners on racial disparities of HIV risk [4, 8, 29].

Additionally, Black indexes (vs. non-Black indexes) reported fewer HIV negative partners on PrEP (24.9% vs. 42.7%) and were more likely to not know their HIV negative partner’s PrEP status (62.3% vs. 36.4%), likely contributing to a higher proportion of HIV TPPs with HIV negative partners among Black indexes (vs. non-Black indexes) (32.6% vs. 17.9%). These findings of decreased PrEP use or decreased knowledge of PrEP status among partners of Black indexes suggest the need for increased PrEP access and discussions of PrEP status. Racial disparities in PrEP uptake and adherence are well documented and contribute to overall racial disparities in HIV prevalence [30].

While the sample size of IDUs was too small to accurately estimate the change in odds of an HIV TPP by IDU status, the increased prevalence of IDU among HIV TPP in this sample suggests further investigation in the relationships between IDU and HIV TPP. IDU both directly increases HIV risk through parenteral HIV transmission [31] and may indirectly increase HIV risk by facilitating a more dense and/or high risk network. Increased IDU has been associated with increased sexual network density [28], and IDUs report higher prevalence of sexual risk behaviors such as exchange sex, individual concurrency, and partner concurrency than users of drugs by other routes [21].

### Limitations

This study has a number of limitations. Self-reported measures of index and partner level PrEP and ART use were used. More precise self-report of index PrEP and ART use (e.g. frequency of use vs. binary yes/no) was available with a one-month recall and used to more accurately describe index viremia and/or HIV risk, but partner PrEP and ART use was only asked as a binary yes/no question with a three month recall. Objective measures of PrEP adherence with the same recall period and HIV viral load would improve the internal validity that a serodiscordant partnership or a partnership with an individual of unknown HIV serostatus was or was not an HIV TPP based on viremia or HIV susceptibility. Sex partner concurrency was also measured by index self-report and could be validated with sociometric network data collection in future studies. Additionally, there was high missing or unknown data on partner HIV serostatus, PrEP use, and ART use. While these data may represent true unknown information and the unknown information is itself important to note, it also reduces the internal validity of the findings. Additionally, the cross-sectional approach only allows us to determine associations, and we cannot yet determine causal relationships. Finally, this study population represents MSM from one urban mid-Atlantic city and may not be generalizable to MSM in other settings.

### Public Health Implications and Next Steps

Persistent racial disparities in HIV prevalence and incidence require new and innovative interventions and/or a reimagining of existing interventions with some demonstrated effectiveness. The evidence presented here reinforces the importance of sexual network structure and the context of mixing dynamics in HIV transmission and therefore the need for network-level interventions. Factors such as sex partner concurrency must be assessed within specific partnerships and contextualized within the broader context of the individual’s sexual network in order to assess how certain sexual risk behaviors may increase or decrease HIV transmission potential. For example, an intervention that trained opinion leaders to be peer educators in the house ballroom community resulted in reduced risk behaviors among fellow house members, including reduced condomless anal sex with partners of unknown HIV status [32]. Adapting this intervention to include education on the importance of condom use with partners of unknown HIV status who may be having concurrent sex may increase the effectiveness of this intervention model. The findings of decreased knowledge of partner serostatus among Black indexes suggest network level interventions to reduce stigma and change norms around HIV serostatus disclosure [33, 34] PrEP [35], and HIV testing [36], and network and structural level interventions to make it easier for individuals to, for example, report PrEP [37] or ART use on online dating profiles or access PrEP or ART more easily. Future research should incorporate partner race and measures of relationship quality, such as trust, and structural factors, such as available partner options, to further elucidate racial disparities in HIV transmission potential and factors that may contribute both to HIV serostatus disclosure and inform decisions about sexual risk behaviors with specific partners.

## Data Availability

The data that support the findings of this study are available from the corresponding author upon reasonable request.

## Code Availability

The code that supports the findings of this study are available from the corresponding author upon reasonable request.
